# Myelomeningocele in Slovenia: An 18-Year National Cohort Study

**DOI:** 10.3390/diagnostics16132036

**Published:** 2026-06-29

**Authors:** Peter Spazzapan, Tomaz Velnar

**Affiliations:** 1 Department of Neurosurgery, University Medical Centre Ljubljana, 1000 Ljubljana, Slovenia; peter.spazzapan@kclj.si; 2 Alma Mater University Maribor, 2000 Maribor, Slovenia

**Keywords:** myelomeningocele, spina bifida, hydrocephalus, paediatric neurosurgery, Slovenia

## Abstract

**Background:** Myelomeningocele (MMC) is a severe neural tube defect resulting from primary neurulation failure. Despite advanced multidisciplinary paradigms, long-term morbidity remains substantial. Population-based longitudinal data from small European cohorts are scarce. This study evaluates long-term clinical and functional outcomes within a complete national cohort in Slovenia. **Methods:** A retrospective cohort study was conducted on all children born with MMC in Slovenia between 2007 and 2023. Patients were managed via a centralized, standardized multidisciplinary program. Phenotypic severity was stratified by anatomical lesion levels, and outcomes were assessed using standardized functional measures. **Results:** Over an 18-year period, 32 children were treated (prevalence: ~1 per 10,000 live births; mean follow-up: 13.2 years). All underwent anatomical closure within 24 h of birth. Hydrocephalus developed in 71.8% (*n* = 23), with 65.6% requiring ventriculoperitoneal shunting. Independent ambulation was achieved by 28.1%, while 46.8% were wheelchair-dependent and paraplegic. Neurogenic bladder dysfunction occurred in 87.5%. Subgroup analysis demonstrated that thoracolumbar lesions were significantly associated with lower ambulation rates and higher shunt dependency compared to lumbosacral lesions (*p* < 0.05). Long-term survival was 96.9%. **Conclusions:** This study represents the first comprehensive national analysis of myelomeningocele outcomes in Slovenia. Despite the relatively small number of patients, complete national coverage and centralized multidisciplinary management provide a unique overview of long-term outcomes. The findings demonstrate that outcomes achieved within the Slovenian healthcare system are comparable to those reported internationally, thereby establishing an important national benchmark for future evaluation of preventive measures and evolving treatment strategies.

## 1. Introduction

Myelomeningocele (MMC) arises from a failure of primary neurulation during the third and fourth weeks of gestation, it involves the herniation of meninges and exposed neural placode through a vertebral arch midline defect. The exposure of vulnerable neural structures results in profound, irreversible motor and sensory deficits [1].

The pathogenesis of MMC is not limited to the initial embryological failure. The widely accepted “two-hit hypothesis” proposes that neurological impairment results from two sequential events [2,3]. The first hit is the failure of neural tube closure. The second hit occurs during foetal development, as the exposed neural tissue is subjected to chronic injury from mechanical trauma, toxic components of amniotic fluid, inflammation, and continuous cerebrospinal fluid (CSF) leakage [4]. This progressive injury provides the biological rationale for prenatal surgical repair, aimed at preventing this secondary damage [5].

Genetic susceptibility, coupled with environmental and maternal factors, contributes to the risk of MMC. Polymorphisms in genes involved in folate metabolism, such as methylenetetrahydrofolate reductase (MTHFR), have been consistently implicated [2]. Maternal folate deficiency is the most well-established modifiable risk factor, and periconceptional folic acid supplementation is proven to reduce NTD incidence by 50–70% [6,7]. Despite successful prevention programs, MMC continues to occur, underscoring the complexity of its aetiology [8]. In addition to folate deficiency and genetic susceptibility, several maternal and environmental factors have been associated with an increased risk of neural tube defects. These include pregestational diabetes mellitus, maternal obesity, hyperthermia during early pregnancy, exposure to teratogenic medications such as valproic acid and other antiepileptic drugs, as well as socioeconomic and nutritional factors. The multifactorial nature of MMC pathogenesis explains why cases continue to occur despite widespread folic acid supplementation and prenatal screening programmes [7,8].

Beyond the primary spinal lesion, MMC initiates a cascade of secondary central nervous system anomalies. Chronic intrauterine CSF venting produces intracranial hypotension, drawing hindbrain structures downward into the foramen magnum and leading to a constellation of neural abnormalities defined as the Chiari II complex. This causes an obstruction of physiological CSF pathways, culminating in ventriculomegaly and obstructive hydrocephalus in the majority of affected neonates. Distal organ systems are similarly impaired, manifesting as neurogenic bladder and bowel dysfunction, complex orthopedic deformities, and variable cognitive deficits [1,2,3].

Advances in neonatal neurosurgery, particularly early postnatal surgical closure, have dramatically improved survival rates [9,10,11,12,13]. The publication of the Management of Myelomeningocele Study (MOMS) in 2011 marked a major advance in the treatment of MMC by demonstrating that prenatal surgical repair can reduce the need for cerebrospinal fluid diversion, improve motor outcomes, and decrease the severity of hindbrain herniation. Since then, numerous specialised fetal surgery centres have refined patient selection criteria, surgical techniques, and perioperative care, resulting in improved maternal and fetal safety profiles. Consequently, prenatal MMC repair has become an established treatment option in selected patients at experienced centres worldwide [5]. Regardless of the timing of the initial repair, clinical priorities have shifted towards optimizing long-term functional outcomes and quality of life through comprehensive multidisciplinary care [14].

**Figure 1:**
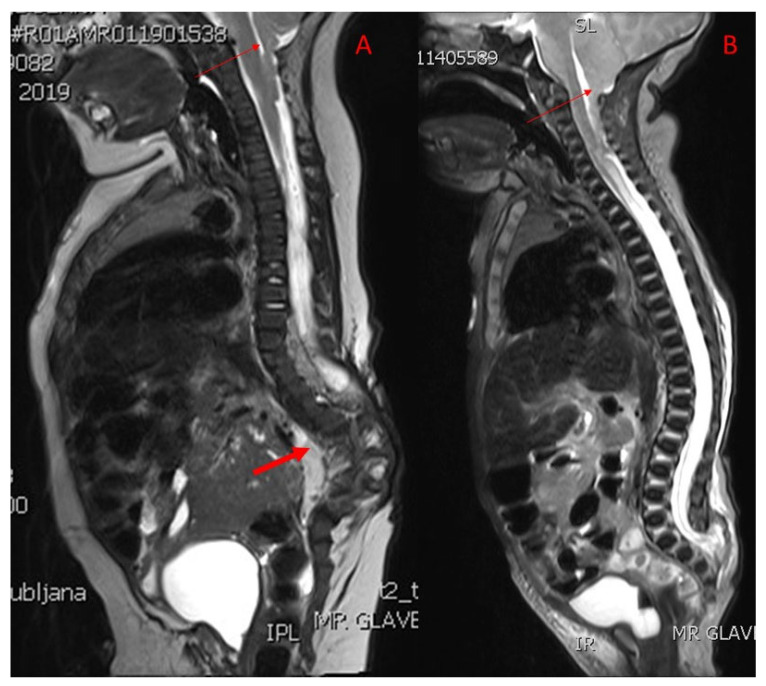
A T2- weighted MRI sagittal image of spine, showing (**A**) extreme lumbar kyphosis (thick arrow) and Chiari II malformation (thin arrow) and (**B**) Chiari II malformation (thin arrow) with pronounced syringobulbia in a previously surgically treated children.

While early postnatal surgical closure has optimized neonatal survival rates, management paradigms have evolved to focus on reducing long-term functional morbidity and maximizing quality of life. Because most international literature originates from large, multi-center registries in highly populated countries, long-term outcome data from complete, well-defined national cohorts in smaller European nations remain sparse. Slovenia possesses a centralized healthcare architecture featuring a single tertiary referral point for all pediatric neurosurgical pathologies, providing a unique opportunity to evaluate an unselected, comprehensive national cohort over an 18-year period. This study evaluates the surgical management, long-term neurological, functional, and systemic sequelae within this cohort, benchmarking the national outcomes against established international standards (Figure 1) [11,12].

## 2. Materials and Methods

This retrospective, population-based cohort study was conducted at the Department of Neurosurgery, University Medical Centre Ljubljana, Slovenia. As the sole tertiary referral center for pediatric neurosurgery in the nation, the institution captures 100% of the country’s live-born MMC cases. Medical records were systematically reviewed for all children born in Slovenia between January 2007 and December 2023 who met the following inclusion criteria: (a) confirmed clinical and radiological diagnosis of myelomeningocele, (b) birth within the geographical borders of Slovenia during the specified 18-year timeframe and (c) completion of continuous, longitudinal follow-up within our multidisciplinary care program.

Patients presenting with closed spinal dysraphisms, lipomyelomeningocele, or isolated meningocele were excluded from the analysis.

**Figure 2:**
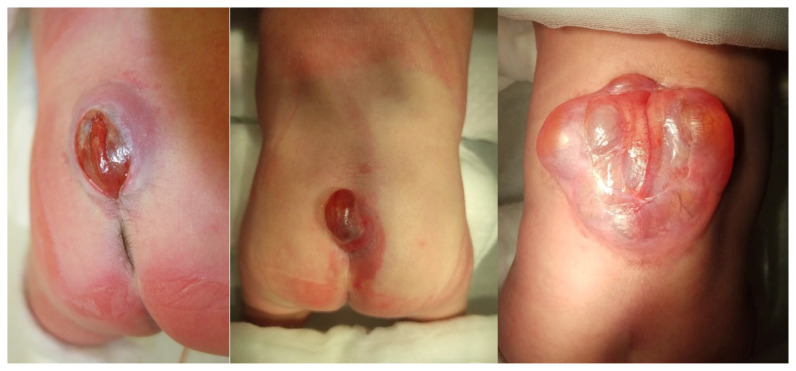
Various forms of spina bifida, encountered at our centre.

**Figure 3:**
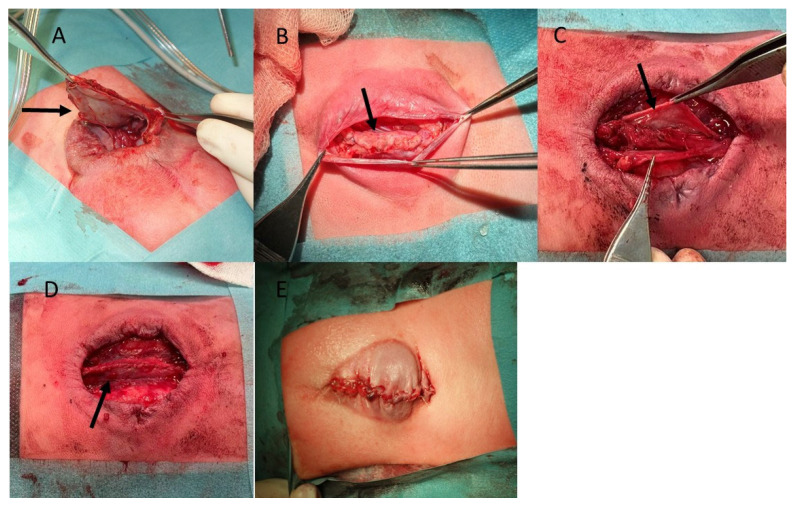
Sequences in surgical treatment of myelomeningocele. (**A**) The placode (arrow) is first carefully dissected from the surrounding tissue and the aberrant nerves are cut. (**B**) The neurulation follows and the neurulated placode (arrow) is closed in a dural sac, after the dural leaves (arrow) are isolated and dissected form the subcutaneous tissue and paraspinal muscles (**C**). (**D**) When dura is closed in a watertight manner (arrow), the muscles and subcutaneous tissue are mobilised and the skin is finally closed (**E**).

All included neonates underwent primary postnatal surgical closure within the first 24 h of life (Figure 2 and Figure 3). The standardized microsurgical protocol involved (a) a careful micro-dissection and untethering of the neural placode from the surrounding epithelial borders, (b) neurulation of the placode, (c) watertight reconstruction and closure of the dural sac, and (d) mobilization of paraspinal myofascial planes to provide a robust mechanical layer, followed by anatomical subcutaneous and cutaneous reconstruction.

Uniform perioperative antibiotic prophylaxis and standardized neonatal intensive care protocols were enforced across the entire study period. Following acute discharge, all children were enrolled into a longitudinal monitoring protocol. Follow-up assessments involved coordinated evaluations by pediatric neurosurgeons, radiologists, paediatricians, neurologists, urologists, orthopedic surgeons, endocrinologists, and physical therapists.

Patients were stratified into two structural subgroups: Thoracolumbar (lesions extending at or above the L2 vertebra) and Lumbosacral (lesions localized strictly at or below the L3 vertebra). The anatomical lesion level was defined by the highest vertebral segment exhibiting open neural arch architecture on pre-operative spinal magnetic resonance imaging (MRI) and confirmed intraoperatively.

Functional ambulation was classified using clinical mobility definitions:

1. Independent Ambulation: Ability to walk continuously without orthoses or handheld assistive devices.
2. Assisted Ambulation: Dependent on ankle-foot orthoses, reciprocal gait orthoses, crutches, or walkers for functional locomotion.
3. Wheelchair Dependence: Completely non-ambulatory, paraplegic state requiring manual or powered wheelchair mobility.

Neurocognitive Function: Cognitive impairment was graded based on standardized IQ intelligence scales during standardized neuropsychological follow-up. Impairment was defined as an IQ < 70 or a documented requirement for specialized institutional educational curricula.

Neurogenic Bladder and Bowel: Evaluated via urodynamic evaluations, renal ultrasonography and clinical tracking of continence or patterns of clean intermittent catheterization.

Data were analyzed utilizing SPSS Version 28.0. Continuous variables are reported as means with corresponding ranges, while categorical variables are expressed as absolute frequencies and percentages. To explore clinical associations between the anatomical lesion level and key secondary endpoints (hydrocephalus, ambulation status, and neurocognitive impairment), Fisher’s exact test was applied to subgroups. Statistical significance was defined as *p* < 0.05.

## 3. Results

A total of 32 children met all eligibility parameters over the 18-year study period, reflecting a national prevalence of approximately 1 case per 10,000 live births in Slovenia. The mean duration of clinical follow-up was 13.2 years (range: 2.5 to 18.0 years). All 32 patients (100%) successfully underwent microsurgical reconstruction within 24 h of birth; perioperative 30-day mortality was 0%. Anatomical stratification revealed that 13 patients (40.6%) presented with thoracolumbar lesions, while 19 patients (59.4%) exhibited lumbosacral defects. Table 1 delineates the distribution of secondary neurological and functional complications stratified by the primary anatomical lesion level.

Analytical evaluation demonstrated a significant association between a higher anatomical defect and worse motor outcomes. Specifically, 84.6% of children in the thoracolumbar subgroup presented with wheelchair dependent paraplegia, compared to only 21.1% in the lumbosacral subgroup (*p* = 0.001). Independent ambulation was entirely restricted to the lumbosacral cohort (47.4% vs. 0.0%, *p* = 0.003). Furthermore, the requirement for ventriculoperitoneal shunting (VPS) was significantly higher in patients with thoracolumbar defects than those with lumbosacral defects (84.6% vs. 52.6%, *p* = 0.046).

Progressive hydrocephalus was diagnosed in 23 children (71.8%) across the entire cohort. VPS served as the primary treatment modality (*n* = 21; 65.6%), whereas 2 patients (6.2%) with favorable anatomy were successfully managed with endoscopic third ventriculostomy (ETV). Late neurosurgical revisions were driven by specific secondary pathologies: 3 patients (9.3%) required secondary surgical untethering for symptomatic tethered cord syndrome, and 4 patients (12.5%) required craniocervical decompression for symptomatic progressive hindbrain herniation.

Neurocognitive impairment was identified in 25 patients (78.1%), manifesting as a heightened requirement for specialized educational programs. Comorbid epilepsy was documented in 6 children (18.7%), all of whom required long-term anticonvulsant pharmacotherapy.

Orthopedic comorbidities, neurogenic bladder and bowel were highly prevalent across long-term follow-up (Table 2). The vast majority of the cohort (*n* = 27; 84.3%) developed long-term orthopedic complications. Lower limb deformities (including talipes equinovarus and hip subluxation) were frequent, requiring targeted orthopedic interventions. Scoliosis occurred in 6 patients (18.7%), with one individual undergoing spinal fusion. Two cases had lumbar kyphosis, one of which (3.1%) underwent a kyphosectomy.

Neurogenic bladder was present in 87.5% of cases, and 78.1% experienced neurogenic bowel, necessitating active urological and gastroenterological regimens. Endocrine imbalances, mainly growth hormone deficiencies and precocious puberty, occurred in 31.2% of children.

Over the 13.2-year mean follow-up period, a mortality rate of 3.1% was observed (*n* = 1), resulting from severe systemic complications unrelated to the neurosurgical site or primary hydrocephalus management.

## 4. Discussion

The results from this 18-year national cohort study offer a comprehensive analysis of the long-term clinical trajectory of MMC within a centralized healthcare system. In Table 3 our cohort outcomes are systematically compared against previous major published series.

Our data clearly illustrate how higher lesions dramatically increase the probability of wheelchair-dependent paraplegia (84.6%) and elevate the incidence of shunt-dependent hydrocephalus (84.6%). The statistically significant increase in VPS among thoracolumbar patients further reinforces the concept that high open spinal lesions cause greater caudal venting of CSF, raising the risks of developing a hydrocephalus [1,2,3]. This chronic intrauterine fluid loss worsens hindbrain herniation, which subsequently impairs posterior fossa compliance and disrupts physiological CSF pathways at the craniocervical junction.

The management of hydrocephalus in MMC remains a primary challenge in pediatric neurosurgery, with lifetime shunt-dependence historically viewed as an unavoidable consequence for most patients. In our national cohort, the overall surgical hydrocephalus rate stood at 71.8%, with 65.6% of the total cohort requiring a VPS and 6.2% managed via ETV. McCarthy et al., in a comprehensive nationwide analysis, noted that 76.8% required surgical management for hydrocephalus during their primary admission [8], confirming that our intervention rate is consistent with large-scale populations.

However, the significant long-term morbidity associated with VPS has driven an international push to reduce shunt placement rates. Chakraborty et al. demonstrated that by adopting conservative monitoring protocols, the absolute rate of shunt placement could be safely reduced to 53% [9]. This approach supports a more restrictive strategy particularly in lumbosacral MMC.

When surgical treatment is necessary, identifying the ideal candidate for ETV remains essential. Tamburrini et al. emphasized that while VPS remains the traditional gold standard, ETV provides an elegant alternative [10]. However, it requires careful patient selection due to anatomical changes in the third ventricle and in the aqueduct. Our successful use of ETV in two patients with low-grade ventriculomegaly and favorable anatomy supports this nuanced approach.

Beyond initial hydrocephalus control, long-term neurosurgical care is frequently driven by the progressive features of the Chiari II malformation. In our series, 12.5% of children required secondary craniocervical decompression for progressive, symptomatic hindbrain herniation. Talamonti et al. noted that while structural hindbrain herniation is radiologically present in nearly all MMC cases, clinical progression demands surgical decompression [2,11]. Their long-term data showed that structured suboccipital decompression with duroplasty effectively stabilizes neurological decline, a pattern reflected in the stabilization of our four symptomatic patients.

However, the high prevalence of neurocognitive impairment (78.1%) and neurogenic bladder dysfunction (87.5%) highlights the persistent morbidity associated with MMC, even when optimal perinatal care is provided [4,5,6,7,12,13]. This high cognitive impairment rate reflects the structural neural changes in the Chiari II constellation, the systemic neurodevelopmental impacts of hydrocephalus and recurrent shunt malfunctions. These findings emphasize that long-term cognitive struggles remain common, necessitating individual educational pathways and structured neuropsychological monitoring from early childhood.

Cortical abnormalities also contribute to long-term neurological morbidity, particularly through secondary epilepsy. We observed a comorbid seizure prevalence of 18.7% across the Slovenian cohort, with a higher absolute distribution among individuals with thoracolumbar defects compared to lumbosacral cases. Similar data were described by Karakas et al., who found an overall seizure prevalence of 18.3% and a correlation between epilepsy and higher anatomical lesion levels, structural brain malformations and a history of recurrent VP shunt malfunctions or infections [14].

Also our functional ambulation parameters (28.1% independent, 25.0% assisted) fall cleanly within the expected international distributions [4,5,6,7,15], confirming that early micro-surgical closure coupled with consistent physical rehabilitation effectively preserves available motor root function.

The absolute prevalence of MMC captured by our study (1 per 10.000 live births) mirrors contemporary European epidemiological trends [2,3]. This low incidence reflects the effective spread in Slovenia of prenatal ultrasonography, targeted maternal serum screening and periconceptional folic acid supplementation [16,17].

While this successful prevention paradigm reduces the absolute volume of children born with MMC, it inherently yields small patient cohorts for single-country studies. However, because our study captures every documented case within the country over an 18-year period, it represents a complete national cohort with high internal validity. This completeness eliminates selection bias and offers a reliable assessment of the performance of a centralized healthcare model.

While this study benefits from complete national coverage and an extensive follow-up duration, several limitations should be noted. Its retrospective nature relies entirely on accurate electronic medical documentation, which restricted our ability to track changes over time using validated, patient-reported quality-of-life instruments or standardized independent functional indices.

## 5. Conclusions

Despite major advances in perinatal neurosurgical techniques and multidisciplinary care, myelomeningocele remains a complex, congenital condition that causes substantial, lifelong neurological and systemic morbidity. Subgroup analysis demonstrates that higher anatomical lesions carry a significantly greater risk of wheelchair-dependent paraplegia and shunt-dependent hydrocephalus.

Our long-term data show that a centralized, single-center management approach in Slovenia achieves low mortality rates and functional outcomes that compare favorably with major international cohorts. However, the high prevalence of long-term neurocognitive, urological, and orthopedic complications emphasizes that early surgical closure is merely the first step in a lifelong management process. Optimizing quality of life and independence for these patients requires a sustained commitment to periconceptional prevention, along with structured, lifelong multidisciplinary support.

## Figures and Tables

**Table 1:** Subgroup analysis of outcomes stratified by anatomical lesion level.

Clinical Endpoint	Total Cohort (*n* = 32)	Thoracolumbar Subgroup (*n* = 13)	Lumbosacral Subgroup (*n* = 19)	*p*-Value
Hydrocephalus Requiring Shunt or ETV	23 (65.6%)	11 (84.6%)	10 (63.1%)	0.046
No Hydrocephalus	9 (28.1%)	2 (15.4%)	7 (36.8%)	0.178
Independent Ambulation	9 (28.1%)	0 (0.0%)	9 (47.4%)	0.003
Ambulation with Assistive Devices	8 (25.0%)	2 (15.4%)	6 (31.6%)	0.285
Wheelchair Dependent Paraplegia	15 (46.8%)	11 (84.6%)	4 (21.1%)	0.001
Neurocognitive Impairment	25 (78.1%)	12 (92.3%)	13 (68.4%)	0.121
Epileptic Seizures	6 (18.7%)	4 (30.8%)	2 (10.5%)	0.165
Neurogenic Bladder	28 (87.5%)	13 (100.0%)	15 (78.9%)	0.117

**Table 2:** Longitudinal orthopedic and visceral morbidity.

Complication Class	Phenotypic Manifestation	Patient Count (*n*)	Percentage (%)
Orthopedic Deformities	Total	27	84.3%
	Scoliosis	6	18.7%
	Lumbar kyphosis	2	6.2%
	Surgical spinal fusion or kyphosectomy	1	3.1%
Visceral Dysfunction	Neurogenic bladder	28	87.5%
	Neurogenic bowel	25	78.1%
Endocrine Deficits		10	31.2%

**Table 3:** Our cohort outcomes compared against previous major published series.

Clinical Endpoint	Current Study, 2026	Adzik et al., 2011 [4]	Spoor et al., 2019 [5]	Zoghi et al., 2024 [6]	Bowman et al., 2001 [7]
Hydrocephalus Rate	71.8%	82.0%	—	—	—
VPS Rate	65.6%	82.0%	79.0%	51.9%	82.0%
Independent Ambulation	28.1%	21.0%	—	—	15.0%
Assisted Ambulation	25.0%	—	—	—	22.0%
Wheelchair Dependence	46.8%	—	—	27.6%	63.0%
Neurogenic Bladder	87.5%	87.0%	91.0%	51.5%	—
Neurogenic Bowel	78.1%	—	77.0%	—	—
Epilepsy	18.7%	—	15.0%	8.3%	—
Cognitive Impairment	78.1%	—	—	—	—
Overall Mortality	3.1%	—	7.0%	6.4%	24.0%

## Data Availability

The data supporting the findings of this study are available from the corresponding author upon reasonable request.
